# Mechanisms of Self-Regulatory Decline in Accusatorial Interrogations

**DOI:** 10.3390/bs15081125

**Published:** 2025-08-19

**Authors:** Amber Heemskerk, Laura Smalarz, Stephanie Madon, Max Guyll, Yueran Yang

**Affiliations:** 1 School of Interdisciplinary Forensics, Arizona State University, Glendale, AZ 85306, USA; laura.smalarz@asu.edu (L.S.); madon@asu.edu (S.M.); guyll@asu.edu (M.G.); 2 Department of Psychology, University of Nevada, Reno, NV 89557, USA; yuerany@unr.edu

**Keywords:** self-regulation, confessions, police interrogation, self-regulatory decline

## Abstract

Confessions carry substantial weight in criminal investigations, yet little is known about the psychological mechanisms underlying suspects’ confession decisions. This research tested the hypothesis that situational pressures inherent to accusatorial interrogations deplete suspects’ self-regulatory resources, impairing their ability to make rational, self-protective decisions. We examined three potential mechanisms of self-regulatory depletion in accusatorial interrogations: (1) decision-making pressure, (2) fatigue, and (3) depleted self-regulatory reserves. Participants were interviewed about minor (Experiment 1; *N* = 154) or serious (Experiment 2; *N* = 486) prior criminal and unethical behaviors under conditions that manipulated whether they experienced both decision-making pressure and fatigue, fatigue alone, or neither. We operationalized decision-making pressure through a response-contingent consequence structure and fatigue through extended questioning. We measured self-regulatory capacity by assessing time spent on an unsolvable anagram task after the interview. Experiment 2 also manipulated whether participants’ pre-interview self-regulatory reserves were depleted by having some complete the unsolvable anagram task before, as opposed to after, the interview. The results suggested a role of decision-making pressure—alone and in combination with fatigue—in producing self-regulatory depletion but provided no evidence for the effect of experimentally depleted self-regulatory reserves. These findings offer empirical support for theories linking interrogation pressures to self-regulatory decline.

## 1. Introduction

Confession evidence is among the most powerful and persuasive forms of evidence in the criminal justice system. Jurors often view confessions as more compelling than other forms of evidence, including eyewitness testimony and forensic analysis (Kassin & Neumann, 1997; Leo, 2008), even when there is reason to doubt their authenticity (Kassin, 2005). Although confessing typically runs counter to a suspect’s legal self-interest, both guilty and innocent suspects sometimes confess. In fact, false confessions were a contributing factor in over 25% of wrongful convictions overturned by DNA (Innocence Project, 2022) and in approximately 13% of the 3608 exonerations recorded overall (National Registry of Exonerations, 2024), underscoring the critical need to understand the psychological processes that can lead suspects to self-incriminate.

One prominent explanation for why suspects confess is that the accusatorial approach characteristic of traditional custodial interrogations impairs their ability to make rational, self-protective decisions through a process of self-regulatory depletion (Davis & Leo, 2012; Guyll et al., 2019; Madon et al., 2017). Self-regulation refers to the deliberate control of one’s thoughts, emotions, and behavior in pursuit of one or more goals (Baumeister et al., 1998; Baumeister, 2014), encompassing processes such as inhibitory control, persistence, and delay of gratification (Muraven & Baumeister, 2000; Inzlicht et al., 2021). These processes rely on a finite pool of psychological resources that can be exhausted through sustained effort (Muraven et al., 1998). When depleted, individuals are prone to making impulsive, short-sighted decisions and to complying with external pressures (Friese et al., 2009). Meta-analytic findings have supported and refined this limited-resource model of self-regulation by showing that depletion effects are strongest for tasks requiring active, goal-directed self-control (Hagger et al., 2010), conditions that typify custodial interrogations.

The accusatorial interrogation approach is particularly likely to impose these kinds of self-regulatory demands due to its reliance on psychologically coercive tactics such as confrontation, minimization, and the presentation of false evidence (see Miller et al., 2018 for a review of interrogation methods). These tactics demonstrably heighten pressure to confess, particularly among innocent suspects (Kassin et al., 2003), and are associated with higher false confessions rates than alternative approaches, such as information-gathering methods (see Catlin et al., 2024). Accordingly, scholars have long criticized the accusatorial method for eliciting confessions by systematically breaking down suspects’ psychological defenses (Kassin et al., 2025; Kassin & Gudjonsson, 2004; Leo, 2008). According to several theoretical models, this breakdown is attributable to self-regulatory decline. For example, the interrogation-related acute situational suggestibility model (Davis & Leo, 2012) proposes that interrogation weakens suspects’ resistance to confession pressures by depleting their self-regulatory resources over time. The biphasic model of resistance (Madon et al., 2017) posits that suspects are initially mobilized to resist interrogation—akin to a fight-or-flight response—but become increasingly vulnerable to short-sighted decision-making as sustained demands exhaust their self-regulatory resources.

Despite these longstanding critiques and theoretical accounts of how accusatorial interrogation methods may heighten susceptibility to coercive influence, the self-regulatory processes involved in suspects’ confession decisions have received limited empirical attention. Few experimental studies have directly tested whether interrogation tactics erode self-regulatory capacity or examined the distinct psychological pathways through which this decline may unfold. The present research addressed these gaps by investigating three proposed mechanisms of self-regulatory depletion in an interrogation context: (1) decision-making pressure, (2) fatigue, and (3) depleted self-regulatory reserves.

### 1.1. Decision-Making Pressure

One core feature of accusatorial interrogations that may tax suspects’ self-regulatory capacity is the decision-making pressure imposed by their response-contingent consequence structure. In this context, suspects must repeatedly decide whether to confess or to continue to deny their guilt. Critically, denying guilt results in immediate discomfort (i.e., continued aversive questioning), whereas confessing brings short-term relief by ending the aversive questioning but carries the long-term risk of conviction. This contingency structure exploits the well-documented cognitive bias of temporal discounting, wherein individuals disproportionately favor short-term outcomes over long-term consequences (Ainslie, 1975; Seaman et al., 2022). According to the interrogation decision-making model (Yang et al., 2017), such temporal discounting tendencies—as well as factors of the interrogation, such as fatigue—drive suspects’ decisions to confess or deny guilt. Empirical research supports this account: Individuals who were questioned about their prior criminal and unethical behaviors shifted their admissions to avoid immediate consequences, even though doing so increased their risk of incurring a future consequence (Madon et al., 2012). Such short-sighted decision-making is exacerbated when the long-term consequences are temporally distant (Yang et al., 2015), abstract or uncertain (Yang et al., 2019), when suspects are tired (Scherr et al., 2014), or when the interrogation is prolonged (Madon et al., 2013).

Self-regulatory depletion may be a key mechanism underlying suspects’ short-sighted decision-making during custodial interrogation. According to confession models grounded in self-regulation theory, suspects experience sustained decision-making pressure as they resist the short-sighted impulse to confess in exchange for immediate relief (Davis & Leo, 2012; Madon et al., 2017). Over time, this continuous effort to maintain resistance in the face of aversive questioning is theorized to deplete suspects’ self-regulatory resources, leaving them increasingly vulnerable to making impulsive, short-sighted confession decisions. Madon et al. (2017) provided preliminary support for this account, finding that participants repeatedly faced with choosing between short-term gains and long-term goals—due to response-contingent consequences—were more susceptible to suggestive questioning, consistent with the effects of self-regulatory depletion. The present research extends this work by directly testing whether decision-making pressure produces measurable self-regulatory decline and by experimentally isolating this effect from the independent influence of fatigue.

### 1.2. Fatigue

Fatigue is a potential byproduct of accusatorial interrogation methods, which emphasize overcoming suspects’ denials through persistence, repetition, and pressure (Drizin & Leo, 2004; Leo, 1996). Although proponents of these methods do not endorse prolonged interrogations—Inbau et al. (2013) recommend limiting sessions to no more than four hours—their confrontational nature often leads to longer periods of questioning than those observed in information-gathering interviews (Meissner et al., 2014). Notably, suspects interrogated using confrontational tactics tend to become less cooperative and more resistant to interrogation pressures than suspects interviewed using rapport-based approaches (Kelly et al., 2016), consistent with the biphasic model of resistance (Madon et al., 2017). This resistance may then prompt interrogators to continue pressing for admissions, setting in motion a cycle of escalating pressure, prolonged questioning, and mounting psychological fatigue.

Prolonged questioning has been shown to exacerbate short-sighted decision-making, consistent with fatigue-induced self-regulatory decline (Madon et al., 2017). In fact, simply questioning people during their circadian “off-peak” periods (e.g., early morning for night owls) can increase their susceptibility to making self-incriminating decisions (Scherr et al., 2014). Moreover, in an analysis of 125 proven false confessions, 84% came from interrogations lasting more than six hours, with an average duration of 16 h (Drizin & Leo, 2004)—far exceeding the typical length of standard police interrogations and the six-hour threshold widely considered coercive (Blair, 2005; Feld, 2006). Together, these findings suggest that fatigue brought on by extended questioning can both directly deplete self-regulatory resources and may also amplify the deleterious effects of other interrogation pressures, such as decision-making demands (e.g., Madon et al., 2013; Scherr et al., 2014).

### 1.3. Depleted Self-Regulatory Reserves

In contrast to decision-making pressure and fatigue, which build over the course of an interrogation, depleted self-regulatory reserves refer to diminished capacity for self-control prior to the interrogation. Suspects may enter interrogations with already diminished self-regulatory reserves due to a variety of situational factors. Sleep deprivation, emotional distress, and physical discomfort—common experiences surrounding arrest and detention (see Leo, 2009)—can impair cognitive functioning and increase vulnerability to external pressure (Solberg Nes et al., 2009; Tice et al., 2001; Venkatraman et al., 2011), putting suspects at a psychological disadvantage before questioning even begins. For instance, sleep deprivation has been shown to reduce impulse control, increase emotional reactivity, and impair decision-making (Harrison & Horne, 2000), all of which are critical for resisting interrogation pressures. Importantly, sleep deprivation has also been identified as a risk factor for false confessions (Blagrove, 1996; Frenda et al., 2016), further underscoring the heightened vulnerability of suspects who enter interrogations in a cognitively depleted state. Thus, prior depletion of self-regulatory reserves may compound the depleting effects of the interrogation itself, leaving suspects particularly vulnerable to making short-sighted confession decisions.

### 1.4. Current Research

The present research aimed to empirically test whether decision-making pressure, fatigue, and depleted self-regulatory reserves erode self-regulatory capacity in an interrogation context. Using a modified version of the repetitive-question paradigm (Madon et al., 2012), participants were interviewed about their involvement in prior criminal and unethical behaviors under conditions that manipulated these three mechanisms of self-regulatory depletion. Decision-making pressure was operationalized through a response-contingent consequence structure in which denials triggered an immediate consequence (i.e., extended questioning) and admissions carried the possibility of a future consequence (i.e., a meeting with a police officer); fatigue was operationalized through extended questioning during the interview; and depleted self-regulatory reserves were operationalized through attempting to solve unsolvable anagrams prior to the interview. Self-regulatory decline was assessed in two ways. First, in both experiments, we measured persistence on an unsolvable anagram task administered after the interview, with reduced persistence indicating diminished self-regulatory capacity. Second, in Experiment 2, we measured the number of admissions made during the interview as an index of short-sighted decision-making under pressure.

In Experiment 1, we tested whether decision-making pressure and fatigue, alone or in combination, produced self-regulatory decline. During the interview, participants experienced either response-contingent consequences and extended questioning (interrogation condition), extended questioning alone (yoked condition), or neither response-contingent consequences nor extended questioning (control condition). We predicted that participants exposed to both response-contingent consequences and extended questioning would exhibit the greatest self-regulatory decline (i.e., spend the least time attempting the anagrams), followed by participants who experienced only extended questioning, with participants who experienced neither showing the greatest persistence.

In Experiment 2, we examined whether the effects observed in Experiment 1 generalized to a higher-stakes context by asking participants about more serious criminal and unethical behaviors during the interview. Experiment 2 also tested whether depleted self-regulatory reserves at the outset of the interview increased susceptibility to short-sighted decision-making. Crucially, the unsolvable anagram task served a dual role in this experiment: For some participants, it was completed before the interview to experimentally deplete self-regulatory reserves; for others, it was completed after the interview to measure post-interview decline (as in Experiment 1). This dual-function approach aligns with prior research that has used unsolvable anagrams to both induce self-regulatory decline (e.g., Starcke et al., 2017) and measure self-regulatory capacity (e.g., Reynard et al., 2011). This design allowed us to test (1) whether beginning the interview in a depleted state increased susceptibility to interrogation pressures—predicted to result in more admissions in the interrogation condition—and (2) whether the interview itself reduced persistence on the anagram task.

## 2. Experiment 1

### 2.1. Experiment 1 Methods

**Design.** We used a single-factor experimental design in which participants were randomly assigned to one of three interview conditions: interrogation, yoked, or control. These conditions varied in their potential to induce self-regulatory decline. In the interrogation condition, participants’ self-regulatory resources were taxed by two processes: decision-making pressure (i.e., repeatedly deciding to deny or admit guilt in the face of response-contingent consequences) and fatigue (i.e., repetitive questions following each denial). In the yoked condition, participants’ self-regulatory resources were taxed by a single process: fatigue. Specifically, yoked participants answered a series of repetitive questions that were predetermined by their matched counterparts in the interrogation condition and not contingent on their own responses. This procedure ensured equivalent exposure to potential psychological fatigue as a result of extended questioning, enabling us to tease apart the unique effects associated with fatigue and decision-making pressure on self-regulatory decline. In the control condition, participants experienced neither response-contingent consequences nor extended questioning and were, therefore, not expected to experience self-regulatory decline.

**Participants.** Undergraduate students (*N* = 167) were recruited through the university’s research participation system in exchange for course credit. We excluded data from participants who experienced technical or administrative errors with the protocol (*n* = 4) or an interruption during the interview (*n* = 1). Additionally, four interrogation participants and four yoked participants were excluded because they either lacked an assigned match (*n* = 4) or their matched partner was excluded (*n* = 4). The final sample consisted of 154 participants (50 interrogation, 50 yoked, 54 control; *M_age_* = 19.28 years, *SD*_age_ = 1.81; 50.0% female; 81.8% Caucasian, 4.6% Asian, 4.6% African American, 3.9% Latino/a, 0.7% Native American, 6.5% identifying with multiple categories).

### 2.2. Experiment 1 Materials and Measures

**Criminal Behavior Interview.** We used a modified version of Madon et al.’s (2012) repetitive question paradigm. Participants responded to 20 interview questions about their prior criminal (e.g., illegally downloading music) and unethical (e.g., purposefully not returning a borrowed item) behaviors. The questions were developed by Madon et al. (2013) and focused on relatively minor behaviors. Participants in the interrogation condition responded to these questions under a response–consequence structure designed to be analogous to what suspects experience in accusatorial interrogations. Specifically, participants in the interrogation condition experienced an immediate consequence every time they denied a behavior (i.e., responding to a set of 32 repetitive questions specifically designed to be psychologically fatiguing). Participants could avoid these questions by admitting to a behavior but were led to believe that doing so increased the risk of incurring a future consequence (i.e., meeting with a police officer to discuss their interview responses).

To isolate the effects of fatigue from the effects of decision-making pressure, we introduced a yoked condition. Each participant in the yoked condition was matched to a participant in the interrogation condition and exposed to the same sequence of repetitive questions as their counterpart, regardless of their own responses. Unlike interrogation participants, they were not informed of a possible police meeting. As a result, participants in the yoked condition experienced an equally fatiguing interview to their counterparts, but without any response-contingent consequences that could create decision-making pressure.

Participants in the control condition completed the interview without any response-dependent consequences, repetitive questions, or mention of a meeting with a police officer. This condition provided a baseline against which to compare the effects of fatigue combined with decision-making pressure (interrogation condition) and fatigue alone (yoked condition).

**Demographic Measures.** Participants reported demographic information including their age, gender, and racial background.

**Anagram Task.** Following the interview, participants attempted to solve 10 anagrams that were, unbeknownst to them, unsolvable. This task served to measure self-regulatory capacity remaining after the interview (e.g., Friedman & Elliot, 2008; Reynard et al., 2011). Participants were told they could work on each anagram for as long or as little as they wished. The anagrams were presented one at a time on a computer screen, and participants provided their responses on a sheet of paper. Participants could proceed to the next anagram using a ‘Next’ button or end the task at any time using an ‘End’ button. If a participant was still working after 30 min, the experimenter terminated the task. Time spent on the task was automatically recorded by the computer.

**Manipulation Checks.** We included two questions as a manipulation check. Participants were asked to indicate whether the experimenter told them they might need to meet with a police officer to discuss their answers (yes/no). Interrogation participants passed this check if they selected ‘yes’, whereas yoked and control participants passed if they selected ‘no’. Additionally, participants were asked to recall the instructions they received at the start of the study by selecting one of two options: (1) I was told I would be signed up to meet with a police officer to discuss my answers if I said “YES” to the questions on the Criminal Behavior Interview, or (2) I was NEVER told that I might have to meet with a police officer to discuss my answers to the Criminal Behavior Interview. Interrogation participants passed this check if they selected option 1, whereas yoked and control participants passed if they selected option 2.

**Suspicion Check.** Participants were asked whether they believed the study investigated “questions that are not obvious.” If they answered ‘yes,’ they were prompted to describe what they believed was under investigation.

**Exploratory Measures.** We included a variety of exploratory measures^1^ that are beyond the scope of the current research aims and will not be further discussed.

### 2.3. Experiment 1 Procedure

Participants were told that the study was being conducted in partnership with local law enforcement personnel to examine the prevalence of criminal and unethical behaviors among college students. After providing informed consent, participants completed demographic and exploratory individual difference measures. The experimenter then explained that the upcoming interview would include two parts with 20 questions each. We led participants to believe that the interview consisted of 40 questions to reduce the likelihood that participants would experience relief upon completing the interview, which could restore their self-regulatory capacity. To reinforce this aspect of the procedure, a printed Behavior Checklist with 40 items hung on the wall in front of the participant, and they were told that their responses would be marked on the checklist during the interview. In reality, participants only responded to 20 interview questions.

Participants in the interrogation condition were also told the following: *“Every time you answer ‘no’ to one of these questions, you’ll be asked some additional follow-up questions so we can get more information”* and *“If you tend to answer ‘yes’ to the questions I ask you, then I will sign you up to meet with one of the police officers involved in this research to discuss your answers in more detail.”* To enhance the realism of this scenario, the experimenter checked a printed schedule and confirmed that the participant would meet with a fictitious officer, named Officer Schiller, if needed. The experimenter then reiterated: *“So basically, if you answer ‘yes’ a lot, you’ll need to meet with Officer Schiller.”*

Participants in the yoked condition were told the following: “There are a few specific behaviors that we are particularly interested in learning more about. So when we get to those behaviors, you’ll be asked some additional follow-up questions.” To reinforce the non-contingent nature of these follow-up questions, the experimenter visibly marked the numbers for these behaviors on the Behavior Checklist, indicating that these follow-ups were pre-determined. Additionally, yoked participants were not led to expect a possible meeting with a police officer.

Participants in the control condition were simply instructed to answer the interview questions as presented, with no mention of follow-up questioning or a future meeting with a police officer.

All participants were then interviewed by the experimenter, consistent with the procedures outlined for their assigned condition. Following the interview, participants were informed that they would complete the anagram task before continuing to Part 2 of the interview. After completing the anagram task, they were told that the second part of the Criminal Behavior Interview would be skipped due to time constraints. Finally, participants completed exploratory measures and manipulation and suspicion check items before being fully debriefed.

### 2.4. Experiment 1 Results

#### 2.4.1. Experiment 1 Preliminary Analyses

**Manipulation Check.** Participants were flagged as having failed the manipulation check if they responded incorrectly to either of the two manipulation check items. We conducted all analyses both including and excluding participants who failed the manipulation check in the interrogation (*n* = 2), yoked (*n* = 4), and control (*n* = 4) conditions, as well as interrogation or yoked participants whose matched partner failed the check (*n* = 5). Removing these participants had no effect on our findings, so we present analyses using the full sample.

**Suspicion Check.** Two independent coders reviewed participants’ open-ended responses about what they believed was under investigation (*n* = 93) to identify participants who were suspicious about the study’s cover story (i.e., the purpose of the repetitive questions or potential meeting with the police officer). Participants were flagged as suspicious if they suspected that we were investigating how the repetitive questions and/or the meeting with the police officer influenced their behavior^2^. No participants explicitly mentioned self-regulation. Interrater reliability was high, with Cohen’s kappa = 0.76, z = 7.36, *p* < 0.001. All discrepancies (*n* = 9) were resolved by the first author.

We conducted all analyses both including and excluding participants who were flagged as suspicious in the interrogation (*n* = 11), yoked (*n* = 9), and control conditions (*n* = 5), as well as matched pairs of suspicious participants in the interrogation and yoked conditions (*n* = 10). Removing these participants had no effect on our findings, so we present analyses using the full sample.

**Data Transformation.** Preliminary analyses revealed that time spent attempting the anagram task deviated from normality, as indicated by a Shapiro–Wilk test, *W* = 0.91, *p* < 0.001, and elevated kurtosis (3.71). Accordingly, we applied a square-root transformation to the variable prior to analysis, which reduced deviation from normality, *W* = 0.98, *p* = 0.064, kurtosis = 2.66. All inferential statistics reflect the transformed data, although raw means and standard deviations are reported for ease of interpretation. Effect sizes and confidence intervals were computed based on the transformed variable.

#### 2.4.2. Experiment 1 Primary Analyses

To evaluate the predicted pattern of self-regulatory capacity—as measured by time spent attempting to solve the unsolvable anagrams after the interview—we conducted a linear trend analysis, a statistical test used to determine whether values systematically increase or decrease across ordered groups. Specifically, we hypothesized that time spent on the anagram task would increase linearly across the three interview conditions: lowest among interrogation participants (who experienced both response-contingent consequences and extended questioning, reflecting the greatest depletion), higher among yoked participants (who experienced extended questioning alone), and highest among control participants (who experienced neither manipulation), such that interrogation < yoked < control.

The results supported this prediction: Interrogation participants spent the least time attempting the anagrams (*M* = 8.77, *SD* = 6.08), followed by yoked participants (*M* = 10.01, *SD* = 7.58), and then control participants (*M* = 11.80, *SD* = 7.36). The linear trend analysis confirmed this pattern, revealing a significant linear effect, *β* = 0.20, *SE* = 0.09, *t*(151) = 2.27, *p* = 0.025, indicating that self-regulatory capacity increased progressively across conditions, consistent with additive effects of decision-making pressure and fatigue.

To further explore this trend, we conducted pairwise follow-up tests. Comparisons between the interrogation and yoked conditions used paired samples *t*-tests to account for the paired structure of these conditions; comparisons involving the control condition used independent-samples *t*-tests. This approach allowed us to evaluate the effects of decision-making pressure (interrogation vs. yoked), fatigue (yoked vs. control), and their additive effects (interrogation vs. control) on self-regulatory depletion. As shown in Table 1, participants in the interrogation condition spent significantly less time on the anagram task than those in the control condition. However, this effect did not remain significant after applying Holm’s correction for multiple comparisons (Holm, 1979). No other pairwise comparisons reached significance.

### 2.5. Experiment 1 Discussion

Experiment 1 investigated whether decision-making pressure and fatigue, alone and in combination, produce self-regulatory decline. Consistent with our hypotheses, we observed a significant linear trend in self-regulatory capacity remaining after the interview—as indicated by the time participants spent on the anagram tasks—across the three interview conditions, with the lowest self-regulatory capacity in the interrogation condition, intermediate capacity in the yoked condition, and the highest capacity in the control condition. This pattern suggests that both decision-making pressure and fatigue contribute to self-regulatory decline in an interrogation context.

Although the difference between the interrogation and control conditions reflected a medium-sized effect (*d* = 0.46), it did not remain statistically significant after correcting for multiple comparisons. Moreover, the difference between the yoked and control conditions was not statistically significant. It is possible that the magnitude of the effects of decision-making pressure and fatigue were modest under the conditions tested in Experiment 1. Specifically, the relatively minor criminal and unethical behaviors used in this experiment—such as illegally downloading music or failing to return a borrowed item—are relatively low-stakes and, thus, may not have elicited the decision-making pressure needed to produce substantial declines in participants’ self-regulatory resources. Moreover, because the behaviors were commonplace, participants frequently admitted to having committed them, which limited the total number of repetitive questions participants were exposed to. In other words, the limited duration of extended questioning may not have been sufficiently fatiguing to produce detectable self-regulatory decline on its own.

In Experiment 2, we introduced more potent manipulations of decision-making pressure and fatigue by asking about more serious criminal and unethical behaviors during the interview. These questions were intended to raise the psychological stakes of the interview and increase fatigue by eliciting a greater number of denials, thereby increasing participants’ exposure to the repetitive questions. Additionally, we introduced an experimental manipulation intended to deplete some participants’ self-regulatory reserves prior to the interview. Specifically, depleted self-regulatory reserves were induced by having some participants attempt the unsolvable anagram task prior to, as opposed to after, the interview. Thus, the unsolvable anagram task functioned as a pre-interview manipulation to deplete self-regulatory reserves for some participants and as a post-interview measure of self-regulatory decline for others. This approach enabled us to additionally test the hypothesis that depleted self-regulatory reserves at the outset of an interrogation increase susceptibility to making short-sighted confession decisions.

## 3. Experiment 2

### 3.1. Experiment 2 Methods

**Design.** We used a 3 (interview condition: interrogation vs. yoked vs. control) × 2 (anagram timing: before interview vs. after interview) factorial design in which participants were randomly assigned to one of the six experimental conditions. The interview condition factor manipulated decision-making pressure and fatigue in the same manner as in Experiment 1. The anagram timing factor manipulated self-regulatory reserves by varying whether participants completed the unsolvable anagram task before or after the interview. In the anagram-before condition, the task was intended to deplete participants’ baseline self-regulatory capacity prior to the interview. In the anagram-after condition, the task served as a measure of remaining self-regulatory capacity following the interview, as in Experiment 1. This between-subjects design allowed us to isolate the effect of pre-interview depletion on admission rates while retaining the task as a measure of the effects of decision-making pressure and fatigue on post-interview self-regulatory capacity.

**Participants.** Undergraduate students (*N* = 581) were recruited through the university’s research participation system in exchange for course credit. We excluded data from participants who personally knew the experimenter (*n* = 2), had previously participated in the study (*n* = 2), experienced an interruption during the interview (*n* = 4), or experienced technical or administrative errors with the protocol (*n* = 50). Additionally, eight interrogation participants and 29 yoked participants were excluded because either they lacked an assigned match (*n* = 16) or their matched partner was excluded (*n* = 21). The final sample consisted of 486 participants (178 control, 154 interrogation, 154 yoked; *M_age_* = 19.28 years, *SD*_age_ = 2.09; 58.4% female; 86.8% Caucasian, 3.5% Asian, 2.67% African American, 2.9% Latino/a, 0.4% Indian, 0.2% Native American, 3.5% identifying with multiple categories).

### 3.2. Experiment 2 Materials, Measures, and Procedures

The materials, measures, and procedures were identical to those used in Experiment 1 unless otherwise noted.

**Criminal Behavior Interview.** To enhance the effectiveness of the interview manipulation, we used interview questions developed by Madon et al. (2012) that focus on relatively serious criminal (e.g., vandalizing property; assaulting someone with the intent to harm them) and unethical (e.g., plagiarism) behaviors. Seven behaviors were consistent across Experiments 1 and 2, while 13 were unique (see Madon et al., 2013 for the full list of minor and serious behaviors).

**Procedures.** Participants assigned to the anagram-before condition completed the anagram task prior to the interview to experimentally deplete their self-regulatory reserves. In contrast, those assigned to the anagram-after condition completed the anagram task after the interview to assess remaining self-regulatory capacity.

### 3.3. Experiment 2 Results

#### 3.3.1. Experiment 2 Preliminary Analyses

**Manipulation Check.** We conducted all analyses both including and excluding participants who failed either manipulation check item in the interrogation (*n* = 4), yoked (*n* = 9), and control (*n* = 6) conditions, as well as those in the interrogation or yoked condition whose matched counterpart failed the manipulation check (*n* = 9). Removing these participants had no effect on our findings, so we present analyses using the full sample.

**Suspicion Check.** Two independent coders reviewed participants’ open-ended responses about what they believed was under investigation (*n* = 298) using the same methods as in Experiment 1. Again, no participants mentioned investigating self-regulation as a focus of the study. Interrater reliability was high, with only 10 discrepancies between coders (Cohen’s kappa = 0.90, z = 15.5, *p* < 0.001), all of which were resolved by the first author.

We conducted all analyses both including and excluding participants flagged as suspicious in the interrogation (*n* = 31), yoked (*n* = 15), and control conditions (*n* = 9), as well as matched pairs of participants flagged as suspicious in the interrogation and yoked conditions (*n* = 40). Removing these participants had no effect on our findings, so we present analyses using the full sample.

**Data Transformation.** Preliminary analyses using Shapiro–Wilk tests indicated non-normal distributions for both dependent variables: time spent attempting the anagram task, *W* = 0.93, *p* < 0.001, kurtosis = 3.81; and interview admissions, *W* = 0.95, *p* < 0.001, kurtosis = 3.50. We applied square-root transformations to both time spent attempting the anagrams (as in Experiment 1) and interview admissions (consistent with Madon et al., 2012, 2013). These transformations improved normality for both time spent attempting the anagrams, *W* = 0.99, *p* = 0.014, kurtosis = 2.89, and interview admissions, *W* = 0.97, *p* < 0.001, kurtosis = 3.18. All inferential statistics reflect the transformed data, although raw means and standard deviations are reported for ease of interpretation. Effect sizes and confidence intervals were computed based on the transformed variables.

#### 3.3.2. Experiment 2 Primary Analyses

We conducted a series of analyses to evaluate the three hypothesized sources of self-regulatory decline: (1) depleted self-regulatory reserves, (2) decision-making pressure, and (3) fatigue. First, we tested whether completing the unsolvable anagram task prior to the interview—designed to deplete self-regulatory reserves—was associated with increased admissions among participants in the interrogation condition. Second, consistent with Experiment 1, we compared time spent attempting the anagrams across the three interview conditions to test whether response-contingent consequences and extended questioning impaired self-regulatory capacity.

**Effect of Anagram Task Timing on Admissions.** To assess whether diminished self-regulatory reserves increased admissions during the interview, we conducted *t*-tests comparing the total number of admissions among participants who completed the anagram task before the interview (i.e., depleted) versus after the interview (i.e., not depleted) separately within each interview condition. We hypothesized that interrogation participants who completed the anagram task before the interview would admit to more behaviors than those who completed it afterward, reflecting increased vulnerability to making short-sighted decisions. No such difference was expected in the yoked or control conditions, as self-regulation was not relevant to these participants’ admissions.

Contrary to our hypothesis, the number of admissions among interrogation participants did not significantly differ between those who completed the anagram task before versus after the interview, *t*(151.33) = 1.17, *p* = 0.737, *d* = 0.19. As expected, no significant differences emerged as a result of the anagram task order in either the yoked condition, *t*(142.46) = 0.87, *p* = 0.771, *d* = 0.14, or the control condition, *t*(166.58) = 0.67, *p* = 0.771, *d* = 0.10. Full *t*-test results are shown in Table 2.

**Effect of Interview Manipulation on Self-Regulatory Capacity**. Consistent with the approach taken in Experiment 1, we first conducted a linear trend analysis to evaluate the predicted pattern of self-regulatory capacity remaining after the interview, as measured by time spent on the anagram task after the interview. Again, we hypothesized that participants in the interrogation condition, who experienced both response-contingent consequences and extended questioning, would spend the least time attempting to solve the anagrams (reflecting the greatest depletion), followed by participants in the yoked condition, who experienced extended questioning alone, and then control participants, who experienced neither (i.e., interrogation < yoked < control).

The results again support this prediction: Participants in the interrogation condition spent the least time attempting the anagrams (*M* = 8.27, *SD* = 5.68), followed by participants in the yoked condition (*M* = 10.03, *SD* = 6.78), and participants in the control condition (*M* = 10.21, *SD* = 6.07). The linear trend analysis confirmed this pattern, revealing a significant linear effect, *β* = 0.20, *SE* = 0.09, *t*(227) = 2.18, *p* = 0.030, indicating that self-regulatory capacity increased progressively from the interrogation to the yoked to the control condition, as predicted.

As in Experiment 1, we conducted follow-up pairwise tests using a paired samples *t*-test to compare the interrogation and yoked conditions and independent samples *t*-tests for comparisons involving the control condition. Also consistent with the results of Experiment 1, participants in the interrogation condition spent significantly less time on the anagram task than those in the control condition, *t*(152.1) = 2.27, *p* = 0.025, *d* = 0.37, but this effect did not remain significant after applying Holm’s correction for multiple comparisons (see Table 3). No other pairwise comparisons reached significance.

#### 3.3.3. Effect of Interview Manipulation on Self-Regulatory Capacity Across Experiments

Given the comparable methodologies used across the experiments for participants who completed the anagram task after the interview, we combined data from participants in Experiment 1 (*N* = 154) with participants in the anagram-after condition of Experiment 2 (*N* = 230) to increase statistical power. To account for differences in interview content (i.e., minor vs. serious behaviors), we included Experiment (1 vs. 2) as a factor in all analyses. For the paired interrogation and yoked conditions, we conducted a mixed ANOVA with interview condition specified as a within-subjects factor and Experiment as a between-subjects factor, as well as their interaction. For comparisons involving the control condition (i.e., yoked vs. control and interrogation vs. control), we conducted separate between-subjects ANOVAs with interview condition, Experiment, and their interaction as between-subjects predictors.

**Effect of Interview Manipulation on Self-Regulatory Capacity.** Consistent with the patterns reported in both experiments, participants in the interrogation condition spent the least amount of time on the anagrams (*M* = 8.47, *SD* = 5.82), followed by participants in the yoked condition (*M* = 10.02, *SD* = 7.08), and then participants in the control condition (*M* = 10.85, *SD* = 6.64). As shown in Table 4, analyses of the full sample yielded a significant effect only for the comparison of participants in the interrogation and control conditions. However, when excluding suspicious participants, there was also a significant effect for the comparison of participants in the interrogation and yoked conditions—pointing to a unique role of decision-making pressure over and above the effect of fatigue, *F*(1,87) = 4.52, *p* = 0.036, *η_p_*^2^ = 0.05. All other findings were consistent when excluding suspicious participants. Together, these findings suggest that the effect of decision-making pressure alone, as well as the additive effects of decision-making pressure and fatigue, depleted self-regulatory capacity.

### 3.4. Experiment 2 Discussion

Experiment 2 was designed to replicate the findings from Experiment 1 using higher-stakes criminal and unethical behaviors and, in addition, to test whether depleted self-regulatory reserves increase suspects’ vulnerability to making short-sighted confession decisions. We predicted that participants in the interrogation condition who attempted unsolvable anagrams before the interview (which presumably depleted their self-regulatory reserves) would exhibit increased admissions, with these higher admission rates serving as our measure of short-sighted decision-making. No such effect was expected in the yoked or control conditions, where short-sighted decision-making was irrelevant given the absence of immediate consequences for denials. Our results did not support this hypothesis. Given the strong theoretical foundation for predicting a role of self-regulatory reserves in suspects’ confession decisions (e.g., Madon et al., 2012, 2013; Yang et al., 2015, 2019), we speculate that this null effect may reflect the limited potency of the anagram task as a depletion manipulation, which we discuss further in Section 4.

Notably, Experiment 2 replicated the Experiment 1 findings regarding the role of decision-making pressure and fatigue in self-regulatory capacity remaining after the interview. Specifically, the linear trend of self-regulatory capacity was again observed in Experiment 2, this time using higher-stakes criminal and unethical behaviors. Moreover, combined analyses of data from Experiments 1 and 2 revealed that participants in the interrogation condition exhibited lower levels of self-regulatory capacity after the interview than those in both the yoked condition (when excluding suspicious participants) and the control condition. These findings suggest that decision-making pressure alone and in combination with fatigue can erode suspects’ self-regulatory capacity. Although the raw differences in self-regulatory capacity remaining after the interview were modest—ranging from approximately 90 to 143 s across conditions in the combined data—they are noteworthy given that such effects are likely to be amplified in real interrogations involving vulnerable populations and/or coercive interrogation tactics, as discussed further in Section 4.

Surprisingly, the effect sizes observed in Experiment 2 (e.g., *d* = 0.37 for interrogation vs. control) were smaller than those observed in Experiment 1 (e.g., *d* = 0.46 for interrogation vs. control), and the effect of extended questioning alone (i.e., yoked vs. control) was again non-significant. We had reasoned that the higher-stakes context and more frequent repetitive questions in Experiment 2 would have imposed greater self-regulatory demands on the participants. However, it is possible that the prospect of meeting with a police officer to discuss their answers to more serious criminal behaviors mobilized participants to resist interrogative pressures, consistent with the biphasic model of resistance (Madon et al., 2017). Additionally, although only participants in the interrogation condition were exposed to explicit response-contingent consequences designed to induce decision-making pressure, participants in all conditions may have experienced some decision-making pressure when asked to admit to potentially embarrassing, unethical, or criminal behaviors during a study they believed was conducted in partnership with local police. This potential for baseline decision-making pressure across conditions may have attenuated differences between groups, potentially underestimating the impact of interrogation-specific pressures. Even so, the pattern of results supports the conclusion that the cumulative demands of accusatorial interrogation pressures can erode suspects’ self-regulatory resources.

## 4. General Discussion

Scholars have proposed that situational forces preceding and during custodial interrogation can deplete suspects’ self-regulatory resources, impairing their ability to make rational, self-protective decisions (Davis & Leo, 2012; Guyll et al., 2013; Madon et al., 2017). In the present research, we evaluated this claim in a controlled laboratory setting, which allowed us to isolate the psychological processes theorized to shape suspects’ confession decisions. Specifically, we investigated three distinct mechanisms through which accusatorial interrogations may erode self-regulatory capacity: (1) decision-making pressure, (2) fatigue, and (3) depleted self-regulatory reserves.

Our findings provided consistent evidence that decision-making pressure and fatigue jointly contribute to self-regulatory decline during custodial interrogation. Participants who experienced both response-contingent consequences and extended questioning evidenced the greatest self-regulatory decline, followed by participants who experienced extended questioning alone. This pattern held across both a low-stakes context involving relatively minor criminal and unethical behaviors (Experiment 1) and a higher-stakes context involving more serious behaviors (Experiment 2). Analyses combining data across both experiments confirmed the additive effect of decision-making pressure and fatigue and provided some evidence of a unique effect of decision-making pressure beyond that of fatigue (i.e., when suspicious participants were excluded). We did not find support for the role of depleted self-regulatory reserves, however, as indicated by a non-significant effect of anagram timing on admissions among participants in the interrogation condition. We discuss potential explanations for this unexpected result below.

### 4.1. Theoretical and Applied Contributions

The findings of this research make several important theoretical contributions. First, they provide direct empirical support for theories of self-regulatory decline in accusatorial interrogations (Davis & Leo, 2012; Madon et al., 2017), which propose that situational forces preceding and during custodial interrogation can deplete suspects’ self-regulatory resources, impairing their ability to make rational, self-protective decisions (Davis & Leo, 2012; Guyll et al., 2013; Madon et al., 2017). The present research offered a direct experimental test of this proposition under controlled laboratory conditions—an approach that affords high internal validity and is well suited to isolating the psychological processes underlying suspects’ confession decisions. Consistent with these theories, our results demonstrated that decision-making pressure and fatigue can significantly erode self-regulatory capacity in the context of confession decisions. These results also align with broader models of self-regulation as a limited capacity resource that can be depleted through sustained cognitive, emotional, or behavioral demands (Baumeister et al., 1998; Muraven & Baumeister, 2000).

Second, our findings highlight the critical role of context in determining when and how self-regulatory decline occurs. Specifically, we observed clear evidence of self-regulatory decline in both low-stakes (Experiment 1) and higher-stakes (Experiment 2) contexts that simulated interrogation pressures but not in response to our experimental manipulation of depleted self-regulatory reserves (i.e., unsolvable anagram task preceding the interview). Given the strong theoretical basis for this effect (Davis & Leo, 2012), the null result of the anagram timing manipulation may reflect a limitation of the anagram task as a depleting mechanism rather than a limitation of the theory itself. According to the process model of self-control (Inzlicht & Schmeichel, 2012), individuals may selectively conserve their self-regulatory resources for tasks that are perceived as meaningful or consequential. This perspective aligns with our findings, which suggest that participants expended their resources when navigating decisions with consequences but not when attempting to solve unsolvable anagrams. While the anagram task may have lacked the motivational salience needed to induce depletion, it nonetheless served as a sensitive measure of self-regulatory capacity remaining after a meaningfully depleting task—the Criminal Behaviors Interview. Together, these findings contribute to the literature on self-regulation by demonstrating that self-regulatory decline is more reliably observed in personally consequential contexts—such as interrogations—than in inconsequential laboratory tasks. This distinction underscores the need for future research to more systematically consider the motivational context of depletion tasks when testing theories of self-regulation.

Third, our findings extend understanding of the psychological mechanisms underlying false confessions by providing direct evidence that decision-making pressure and extended questioning can systematically diminish suspects’ self-regulatory resources. This finding supports frameworks suggesting that situational pressures deplete suspects’ regulatory resources, thereby increasing susceptibility to short-sighted confession decisions (Davis & Leo, 2012; Madon et al., 2017). Our findings also complement the interrogation decision-making model (Yang et al., 2017), which posits that suspects’ decisions to confess or deny guilt are guided by a dynamic cost–benefit analysis that accounts for the cumulative effects of fatigue, interrogation techniques, and the response-contingent consequence structure. Indeed, self-regulatory decline may be a central mechanism through which such situational factors impair suspects’ ability to make rational, self-protective choices. More broadly, these situational contingencies can also be interpreted through a behaviorist lens. Our design’s use of prolonged questioning following denials—a form of positive punishment—mirrors real-world interrogation practices (Niland & Ortu, 2020). From this perspective, regulatory decline may reduce suspects’ resistance to reinforcement schedules, thereby increasing their susceptibility to operant conditioning. Together, these complementary frameworks offer a more comprehensive account of how interrogation tactics shape confession decisions.

The current research also offers new insights regarding recommended interrogation reforms. In particular, the findings suggest that interrogation practices that minimize unnecessary strain on suspects’ self-regulatory resources (see Meissner et al., 2014) may help reduce the risk of impaired decision-making. Accusatorial interrogations are adversarial by design and can entail coercive pressure and the strategic manipulation of perceived consequences (Kassin et al., 2010). These tactics may heighten innocent suspects’ vulnerability to impaired decision-making by imposing a substantial self-regulatory cost. Over time, the cumulative self-control demands imposed by the interrogation may impair suspects’ ability to resist coercive pressure to confess (e.g., Guyll et al., 2019; Madon et al., 2017), potentially increasing the likelihood of a false confession (e.g., Madon et al., 2012). Thus, our findings raise the possibility that self-regulatory decline could increase a suspect’s vulnerability to coercive interrogation tactics. This vulnerability underscores the relevance of recommendations outlined in the recent Scientific Review Paper on Police-Induced Confessions (Kassin et al., 2025), which calls for limiting the duration of interrogations, avoiding minimization themes that imply leniency for confessions, and restricting the presentation of false evidence that makes continued denials seem futile.

### 4.2. Limitations and Future Directions

The results of this research should be viewed in light of several limitations. First, the generalizability of our findings to custodial interrogations is constrained by our reliance on relatively minor behaviors and consequences. Although we attempted to increase the stakes of the interview context in Experiment 2 by incorporating more serious behaviors, there are elements of custodial interrogations that are difficult to emulate in a laboratory setting. Nevertheless, because participants in the interrogation condition were interviewed under the presumption that their admissions could have real long-term consequences (a future meeting with a police officer) and the extended questions were tedious by design, we believe that our operationalizations of decision-making pressure and extended questioning tapped psychological processes analogous to those that operate to deplete suspects’ self-regulatory capacity during custodial interrogations. Thus, our results may reflect the levels of self-regulatory decline occurring during short and/or low-pressure custodial interrogations. In lengthy interrogations and those characterized by more potent situational pressures, we would expect to observe substantially larger self-regulatory decline effects than those observed here.

Second, our sample consisted exclusively of college students, who do not fully represent the broader population of suspects subjected to custodial interrogation. Vulnerable populations—such as adolescents, individuals from marginalized backgrounds, and those with mental health conditions—are overrepresented in the criminal justice system and may be particularly susceptible to interrogation-related self-regulatory decline (see Najdowski, 2011; Redlich & Goodman, 2003). Future studies should prioritize more diverse and representative samples, with particular attention to vulnerable populations, to better understand the potential of custodial interrogation to deplete self-regulatory resources in those groups.

Third, while our design isolated one well-established component of self-regulation—persistence on a difficult task (e.g., Hagger et al., 2010; Muraven et al., 1998)—it did not capture the full breadth of self-regulatory functioning. Therefore, it is possible that a more comprehensive measure of self-regulation might have revealed stronger effects than those observed here. For example, interrogation-related pressures may have a greater impact on domains such as impulse control, emotional regulation, or higher-order reasoning than on task persistence. The null effect of the anagram order manipulation may also reflect the limited effectiveness of the unsolvable anagram task as a depleting mechanism. This interpretation aligns with growing concerns about the reliability of laboratory-based depletion paradigms, particularly when the initial task lacks personal relevance or motivational weight (Hagger et al., 2016; Inzlicht & Schmeichel, 2012). It is plausible that the unsolvable anagram task was not sufficiently motivating or consequential to induce meaningful self-regulatory depletion. Future research should explore more ecologically valid methods of pre-interrogation depletion—such as sleep deprivation (e.g., Harrison & Horne, 2000), emotionally distressing events (e.g., Tice et al., 2004), or cognitively taxing deception detection interviews (e.g., Vrij et al., 2008)—which may more accurately capture the cognitive and emotional load suspects experience prior to interrogation.

## 5. Conclusions

A large body of research supports the limited-resource model of self-regulation (Dang et al., 2021), but the boundaries of this effect have been called into question (Hagger et al., 2010). Separately, the role of false confessions in known wrongful convictions has raised critical concerns about how accusatorial interrogations may impair suspects’ rational decision-making (Kassin et al., 2025). This study aimed to integrate these lines of work by examining three potential mechanisms of self-regulatory depletion in accusatorial interrogations: decision-making pressure, fatigue, and depleted self-regulatory reserves. Across two experiments, we found consistent evidence that decision-making pressure—both alone and in combination with fatigue—impairs self-regulatory capacity, as measured by persistence on a difficult task. However, depleted self-regulatory reserves did not significantly increase the likelihood of admissions. Future research should employ more ecologically valid pre-interrogation depletion methods to assess their effect on confession decisions. Overall, these findings offer novel insight into how accusatorial interrogations may erode suspects’ capacity for rational, self-protective decision-making and highlight the importance of contextual factors in self-regulatory depletion.

## Figures and Tables

**Table 1:** Comparisons of time spent attempting anagrams between interview conditions in Experiment 1.

	Mean			*p*			
Comparison	Difference	*df*	*t*	Raw	Adj.	*d*	95% CI
Interrogation vs. Yoked	−1.24	49	−0.78	0.438	0.438	−0.11	[−0.39, 0.17]
Yoked vs. Control	−1.79	100	−1.43	0.157	0.313	−0.28	[−0.67, 0.11]
Interrogation vs. Control	−3.03	102	−2.35	0.021	0.063	−0.46	[−0.85, −0.07]

*Note*. Mean differences are presented in raw units. Degrees of freedom (*df*), *t* values, *p* values (raw and Holm-adjusted), Cohen’s *d*, and 95% confidence intervals (CIs) for Cohen’s *d* are based on the transformed data.

**Table 2:** Mean admissions across experimental conditions in Experiment 2.

	Anagrams Before	Anagrams After			*p*			
Condition	*M* (*SD*)	*M* (*SD*)	*df*	*t*	Raw	Adj.	*d*	95% CI
Interrogation	4.38 (3.34)	5.12 (4.10)	151	–1.17	0.246	0.737	–0.19	[–0.50, 0.13]
Yoked	4.46 (2.61)	4.28 (3.14)	142	0.87	0.385	0.771	0.14	[–0.18, 0.46]
Control	4.73 (2.75)	4.46 (2.96)	167	0.67	0.506	0.771	0.10	[–0.19, 0.40]

*Note*. Means and standard deviations, *M* (*SD*), are presented in raw units. Degrees of freedom (*df*), *t* values, *p* values (raw and Holm-adjusted), Cohen’s *d*, and 95% confidence intervals (CIs) for Cohen’s *d* are based on the transformed values.

**Table 3:** Comparisons of time spent attempting anagrams post-interview between interview conditions in Experiment 2.

	Mean			*p*			
Comparison	Difference	*df*	*t*	Raw	Adj.	*d*	95% CI
Interrogation vs. Yoked	–1.76	74	−1.86	0.067	0.133	−0.22	[−0.44, 0.01]
Yoked vs. Control	−0.18	148	−0.43	0.670	0.670	−0.07	[−0.38, 0.25]
Interrogation vs. Control	−1.94	152	−2.27	0.025	0.074	−0.37	[−0.68, −0.05]

*Note*. Means and standard deviations, *M* (*SD*), are presented in raw units. Degrees of freedom (*df*), *t* values, *p* values (raw and Holm-adjusted), Cohen’s *d*, and 95% confidence intervals (CIs) for Cohen’s *d* are based on the transformed values.

**Table 4:** Effect of interview condition on time spent attempting anagrams for Experiments 1 and 2 combined.

Predictor	*df*	*MSE*	*F*	*p*	*η_p_* ^2^	95% CI
Interrogation vs. Yoked						
Interview Condition	1, 123	0.95	3.08	0.082	0.02	[0.00, 0.09]
Experiment	1, 123	1.16	0.01	0.916	0.00	[0.00, 0.01]
Interview Condition × Experiment	1, 123	0.95	0.17	0.682	0.00	[0.00, 0.03]
Yoked vs. Control						
Interview Condition	1, 255	1.07	2.08	0.151	0.01	[0.00, 0.04]
Experiment	1, 255	1.07	0.41	0.520	0.00	[0.00, 0.02]
Interview Condition × Experiment	1, 255	1.07	0.85	0.356	0.00	[0.00, 0.03]
Interrogation vs. Control						
Interview Condition	1, 255	0.96	10.91	0.001	0.04	[0.01, 0.09]
Experiment	1, 255	0.96	1.20	0.274	0.00	[0.00, 0.03]
Interview Condition × Experiment	1, 255	0.96	0.32	0.575	0.00	[0.00, 0.02]

*Note*. Degrees of freedom (*df*) for the effect and error terms, mean square error (*MSE*), *F* value, *p* values, partial eta-squared (*η_p_^2^*), and 95% confidence intervals (CIs) for *η_p_*^2^ are reported based on the transformed values.

## Data Availability

The original data and supporting materials for this study are openly available on Open Science Framework at https://osf.io/pcgyv/ (accessed 27 May 2025).

## Notes

1 Exploratory measures included a variety of individual difference measures and items assessing participants’ perceptions of the anagram task (e.g., “*The anagrams were stressful*”), repetitive questions (e.g., “*If you could have, how much would you have liked to have skipped the additional questions?*”), and the potential meeting with a police officer (e.g., “*Assuming that you are contacted for a meeting with the police officer, how compelled would you feel to go through with it?*”). Additionally, participants were asked to honestly report their answers to the Criminal Behavior Interview privately on a computer. All exploratory measures and data are available on Open Science Framework (OSF).

2 Although only participants in the interrogation and yoked conditions were exposed to these manipulations, suspicious participants were identified in all three conditions. We suspect that the manipulation check items, which preceded the suspicion probe, may have inadvertently cued some participants to the purpose of the research.
